# *Withania coagulans*-mediated green synthesis of silver nanoparticles: characterization and assessment of their phytochemical, antioxidant, toxicity, and antimicrobial activities

**DOI:** 10.1186/s12870-025-06533-7

**Published:** 2025-05-02

**Authors:** Amjid Khan, Tahira Younis, Muhammad Anas, Muhammad Ali, Zabta Khan Shinwari, Ali Talha Khalil, Khurram Shahzad Munawar, Hamza Elsayed Ahmed Mohamed, Khaoula Hkiri, Malik Maaza, Mahmoud F. Seleiman, Naeem Khan

**Affiliations:** 1 Department of Plant Sciences, Faculty of Biological Sciences, Quaid -i- Azam University, Islamabad, 45320 Pakistan; 2 Department of Botany, University of Mianwali, Mianwali, Punjab, 42200 Pakistan; 3 Department of Biotechnology, Faculty of Biological Sciences, Quaid-i-Azam University, Islamabad, 45320 Pakistan; 4 Federal Urdu University of Arts, Sciences and Technology, Karachi, 75300 Pakistan; 5 Department of Pathology, Lady Reading Hosipital Medical Teaching Institution, Peshawar, KP 25000 Pakistan; 6 Department of Chemistry, University of Mianwali, Mianwali, Punjab, 42200 Pakistan; 7 Institute of Chemistry, University of Sargodha, Punjab, 40100 Pakistan; 8 UNESCO UNISA Africa Chair in Nanoscience and Nanotechnology, College of Graduate Studies, University of South Africa, Pretoria, South Africa; 9 Nanoscience African Network (NANOAFNET), Materials Research Department, iThemba LABS, Cape Town, South Africa; 10 Department of Plant Production, College of Food and Agriculture Sciences, King Saud University, Riyadh, Saudi Arabia; 11 Agronomy Department, Institute of Food and Agricultural Sciences, University of Florida, Gainesville, FL 32611 USA

**Keywords:** Antimicrobial activity, Antioxidant, Green synthesis, Nanotechnology, Silver nanoparticles

## Abstract

**Background:**

In this study, we report the biofabrication of silver nanoparticles (Ag-NPs) using aqueous leaf extracts of *Withania coagulans*, which act as both reducing and capping agents. The goal was to synthesize and characterize the silver nanoparticles and evaluate their biological properties.

**Results:**

The silver nanoparticles were characterized by multiple techniques including UV–visible spectroscopy, X-ray diffraction (XRD), Fourier-transform infrared spectroscopy (FTIR), Raman spectroscopy, scanning electron microscopy (SEM), energy-dispersive spectroscopy (EDS), high-resolution transmission electron microscopy (HRTEM), zeta potential, dynamic light scattering (DLS), thermogravimetric analysis (TGA), and differential scanning calorimetry (DSC). A surface plasmon resonance peak was observed at 420 nm, and the XRD pattern indicated highly crystalline Ag-NPs with a crystallite size of 39.76 nm. SEM and HRTEM revealed irregular morphology with an average particle diameter of 26.63 nm. Zeta potential of -21.4 mV indicated relatively stable nanoparticles. FTIR spectra displayed significant peaks at 3269, 2921, 1628, 1513, and 1385 cm^−1^. Thermal stability was confirmed via TGA and DSC. Bioassays including total phenolics, total flavonoids, ferric reducing antioxidant power, and DPPH assays showed higher antioxidant potential in Ag-NPs compared to extracts, though phenolic and flavonoid content was lower. Biocompatibility tests such as hemolysis (*IC*_*50*_ = 141.466 μg/mL) and brine shrimp lethality assay (*IC*_*50*_ = 721.76 μg/mL) indicated moderate cytotoxicity. Phytotoxicity assays revealed higher toxicity of Ag-NPs against radish compared to control. Significant antibacterial activity was observed against *Klebsiella pneumoniae* and *Salmonella typhi* (29 ± 0.01 mm and 28 ± 1.00 mm inhibition zones at 25 μg/mL, respectively).

**Conclusions:**

The *Withania coagulans* leaf-extract-mediated silver nanoparticles exhibit remarkable antioxidant, phytochemical, and antimicrobial properties, suggesting potential for commercial applications in various biomedical and agricultural fields.

## Introduction

Medicinal plants are invaluable sources of unique chemical compounds, with approximately 25% of prescribed drugs in developed nations derived from wild plant species, highlighting their essential role in healthcare and the advancement of traditional medicine [1]. Plants synthesize primary and secondary metabolites, many of which possess substantial therapeutic potential [2, 3]. Tannins, alkaloids, sugars, terpenoids, steroids, and flavonoids represent essential phytochemical components within medicinal plants, contributing significantly to their medicinal attributes [4–7]. The plant we are studying, *Withania coagulans* (L.) Dunal, belongs to the Solanaceae family and is highly valued for its pharmacological properties. Unfortunately, it is currently classified as an endangered plant due to factors such as excessive harvesting, low seed germination rate, reproductive issues, and the presence of unisexual flowers [8, 9]. *W. coagulans* is an herb that is indigenous to both temperate (Afghanistan) and tropical regions (Pakistan, India) of Asia. The presence of more than 12 alkaloids and 40 distinct types of withanolides confers its therapeutic properties [10]. *W. coagulans* has been significantly known in Ayurveda to have several health benefits, including having the potential to prevent diabetes, lower cholesterol levels, reduce oxidative stress, combat cancer, inhibit microorganism growth, scavenge free radicals, and reduce inflammation. Moreover, *W. coagulans* extracts have shown promising effects in wound healing, treating skin disorders, and alleviating gastrointestinal ailments [11, 12].

Nanotechnology involves the precise manipulation and control of materials ranging from 1 to 100 nm in size, with applications spanning medicine, electronics, energy production, and consumer goods [13–16]. Several factors affect the size, shape, and stability of nanoparticles (NPs), including pH, temperature, growth medium, synthesis conditions and surface properties [17–19]. Nanoparticles have gained the attention of researchers in the field of nanomedicine, pharmaceutical, agriculture, targeted drug delivery and their role in biomedical applications [20, 21]. The therapeutic efficacy of nanoparticles depends on by several factors, including the size of the particles, the duration of cell growth, the concentration of metal in the specific cell, and the physicochemical characteristics of the NPs [22, 23]. Green synthesis methods utilizing natural extracts or biomolecules as reducing and stabilizing agents have emerged as viable alternatives to traditional synthetic processes. The environmentally friendly production of metal nanoparticles is garnering significant attention due to their potential applications in photocatalytic dye degradation, electrochemical sensing, biofilm, antioxidant, anti-inflammatory, and anti-diabetic activities, its non-toxicity, cost-effectiveness, environmental friendliness, and commercial viability [24–29]. Biomaterial extracts contain secondary metabolites and bioactive molecules that act as reducing, capping, stabilizing, and chelating agents, replacing harmful chemical surfactants in the nanoparticle formation process [27, 30, 31]. The green method for phytomediated nanoparticle synthesis has gained widespread attention across disciplines, as it enhances the environmental benefits of nanoparticles by using only plant materials, agricultural waste, and enzymes. This approach has been successfully scaled for large-scale production, yielding nanoparticles with distinct shapes and small sizes. Compared to traditional methods, green synthesis is less harmful to the environment, humans, and other living systems [32–35]. While all metallic nanoparticles, including noble metals, are viable, silver nanoparticles (Ag-NPs) stand out for their exceptional effectiveness in various applications. These can be incorporated into fiber composites, cryogenic superconducting materials, electronic components, and products in the cosmetic and food industries. Moreover, Ag-NPs exhibit significant antiviral, antibacterial, antifungal, and anti-inflammatory properties, making them highly sought after. Due to their broad-spectrum biocidal properties against microbes, silver and silver-based compounds are used in wound dressings, topical lotions, and antiseptic sprays. The use of Ag-NPs in these products has shown promising results in wound healing and anti-diabetic applications [36–39]. Among various metals, silver nanoparticles are preferred due to their natural composition and non-toxic nature, ensuring safety for human health [40]. Silver nanoparticles are widely used in the pharmaceutical industry because of their unique physicochemical characteristics, particularly their significantly high surface-to-volume ratio, serving as effective agents with antibacterial, antioxidant, and anticancer capabilities [41–44]. Utilizing biological materials such as plant extracts, bacteria, fungi, algae, chitosan, etc., for Ag-NPs synthesis by green methods shows many advantages. This method uses the beneficial characteristics of silver as a noble metal and enhances the properties of nanoparticles, hence improving their biological applications [45]. Given that the primary component is readily accessible and cost-effective, synthesizing silver nanoparticles through plant-based methods is well-suited for large-scale production. This approach proves to be safe, and environmentally friendly in comparison to the expensive physical and chemical methods of Ag-NPs synthesis, which often involve toxic chemicals that pose risks to both individuals and the environment [46]. Plant-based silver nanoparticles have shown enhanced properties including antioxidant, cytotoxic, anticancer, anti-inflammatory, antibacterial, and antidiabetic activities [47–49].

This study presents the green synthesis of silver nanoparticles using *Withania coagulans* leaf extracts as reducing and capping agents, leveraging the plant’s pharmacologically active compounds in nanoparticle synthesis; a method not extensively explored. The environmentally friendly approach offers a sustainable and cost-effective alternative to conventional methods that rely on toxic chemicals. However, synthesis outcomes may vary with factors like pH, temperature, and environmental conditions, affecting nanoparticle size, shape, and stability. Additionally, the synthesized Ag-NPs have slightly lower flavonoid and phenolic content than crude extracts, which may influence bioactivity. The nanoparticles are characterized through multiple techniques, including UV–Vis, XRD, FT-IR, Raman spectroscopy, SEM-EDS, HR-TEM, zeta potential, DLS, TGA, and DSC. Furthermore, their phytochemical, antioxidant, biocompatibility, phytotoxicity, and antibacterial properties are thoroughly evaluated. While previous studies have synthesized Ag-NPs with various plant extracts, this work provides the first detailed analysis of *Withania coagulans*-mediated Ag-NPs, integrating structural characterization with antioxidant, cytotoxic, and antimicrobial assessments. This combined approach highlights *Withania coagulans* as a promising bioreductant for multifunctional applications in biomedicine and agriculture.

## Materials and methods

### Collection of plant materials

Experimental work was conducted at the Molecular Systematics and Applied Ethnobotany Lab (MOSAEL), Department of Plant Sciences, Quaid-i-Azam University (QAU), Islamabad. The leaves of *Withania coagulans* were collected in June 2022 from Village Sawans, District Mianwali, Punjab Province, Pakistan. The collection was performed on private land with explicit permission from the landowner, ensuring compliance with institutional, national, and international guidelines. Taxonomic identification of the plant material was carried out at the Department of Plant Sciences, QAU, Islamabad, by Prof. Dr. Zabta Khan Shinwari, an expert taxonomist. A voucher specimen (No. 133570) was deposited in the Herbarium of Pakistan (ISL) for documentation and future reference. After collection, the leaves were washed with tap water, shade-dried, and finely ground into powder. The powdered leaves were then mixed with distilled water (dH_2_O; 10 g/100 mL) and stirred for 24 h at room temperature. The extract was filtered using Whatman No. 1 filter paper and stored at 4 °C for further analysis.

### Green synthesis, characterization and mechanism of formation of Ag-NPs using plant extract

**Fig. 1:**
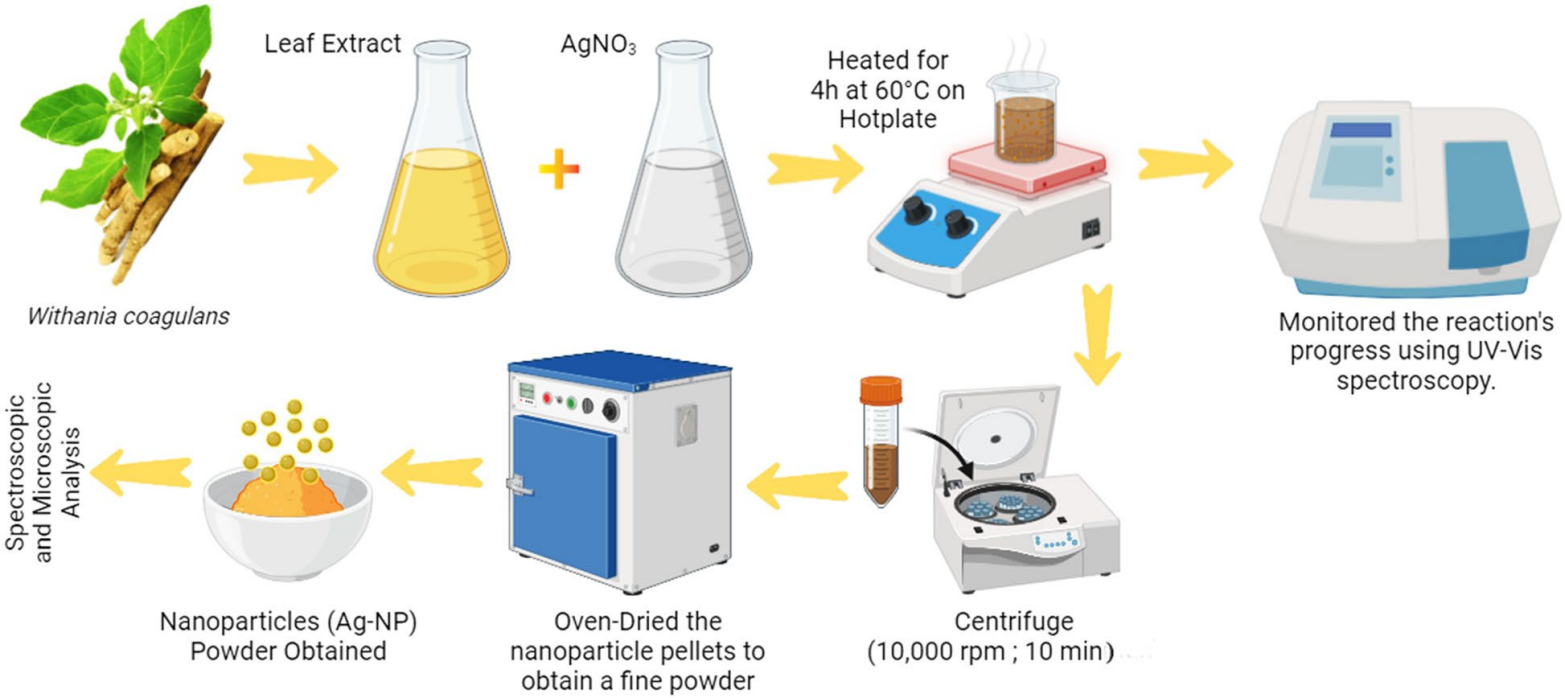
Workflow for green synthesis of Ag-NPs using *W. coagulans* leaf extract. The image was created using biorender

**Fig. 2:**
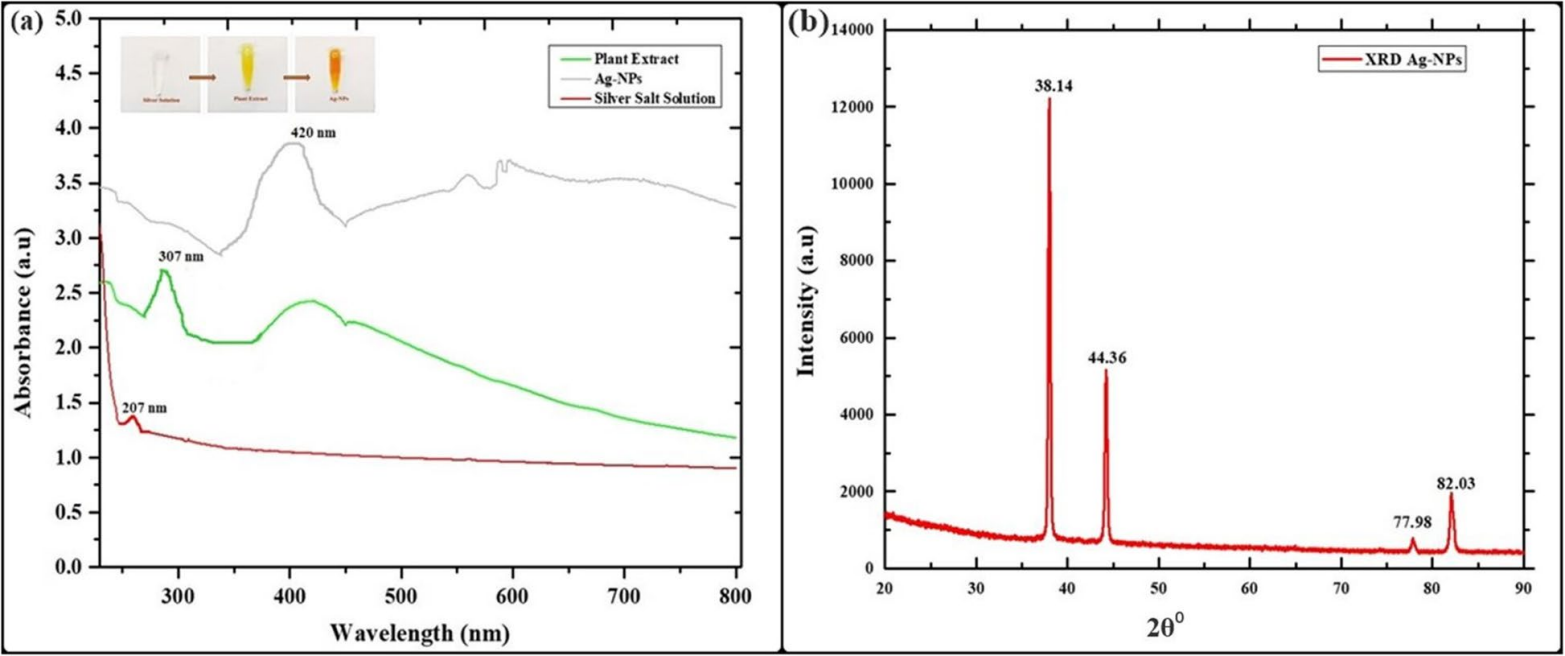
Physicochemical characterization of biosynthesized Ag-NPs. **a** The UV–Vis absorption spectra of the Ag-NPs, **b** X-ray diffraction pattern of synthesized Ag-NPs

The bio-reduction mechanism for forming Ag-NPs using plant extracts involves the trapping of Ag^+^ ions on protein surfaces through electrostatic interactions. These proteins then reduce the Ag^+^ ions, causing structural changes and forming silver nuclei. As the reduction continues, additional Ag^+^ ions accumulate at these nuclei, leading to the gradual growth of Ag-NPs [50]. Another study highlighted that the primary mechanism in plant-mediated Ag-NPs production is phytochemical-assisted reduction, involving key compounds such as ketones, terpenoids, amides, flavones, carboxylic acids, and aldehydes [51]. Silver nanoparticles were produced by combining an aqueous leaf extract of *W*. *coagulans* with an AgNO_3_ solution (AgNO_3_; 1 mmol/L) in a 1:9 ratio. This synthesis process, conducted at 60 °C on a hot plate for 4 h, followed a method with slight adjustments outlined by Sharifi-Rad et al. [52]. The reaction mixture from pale yellow to dark brown, indicating progress, confirmed by UV–Vis spectroscopy. Post-mixture centrifugation at 10,000 rpm for 10 m and based on the precipitates were Ag-NPs, they were washed three times with distilled water, then collected and subjected to oven-drying, yielding a fine powder. This powder was subsequently utilized for various characterizations. XRD (an Empyrean diffractometer from Malvern Panalytical) was used to establish structural and crystalline properties of Ag-NPs. FT-IR spectra was acquired to study the associated functional groups. FT-IR revealed functional groups in natural chemicals that act as reducing, capping, and stabilizing agents. Raman spectra were acquired at room temperature using a laser with a wavelength of 473nm and an average excitation power of 2.48MW. Ultraviolet–visible Spectrophotometry (UV–Vis, UV-2600) identified the SPR resonance peak (Fig. 1). High resolution scanning/transmission electron microscopy (HR-SEM/TEM). analyzed nanoparticle shape and size. Elemental composition was obtained using energy-dispersive spectroscopy (EDS) on a Quanta Feg450 instrument. Dynamic Light Scattering (DLS) evaluates the hydrodynamic diameter of nanoparticles in a solution and offers insights into their clustering status using a ZS 90 particle analyzer and Malvern Instrument (Zetasizer Ver. 8.00.4813) analyzed the zeta potentials. The thermal stability and decomposition rates of the Ag-NPs were evaluated through differential thermal gravimetric analysis (TGA–DSC). Workflow for green synthesis of Ag-NPs using *W. coagulans* leaf extract was shown in Figs. 1 and 2.

### Phytochemical screening and antioxidant activities

#### Total Phenolics Content (TPC) and Total Flavonoids Content (TFC)

The total phenolic content (TPC) of plant extracts and produced Ag-NPs was measured using the Folin-Ciocalteu method with minor changes, according to the methodology by Rahman et al. [53]. This involved using a stock solution of Folin- Ciocalteu reagent and sodium carbonate. The experiment was carried out in a 96-well microplate with gallic acid as the positive control. Absorbance (Abs) values were taken at 630 nm using a Bio-Tek Instruments microplate reader. TPC was measured in mg GAE/g dry weight for both the plant extract and Ag-NPs. Standard calibration was established by evaluating gallic acid standards under identical conditions. The concentration range of the standard solutions was 25–100 µg mL^−1^, with a linear equation of (y = 0.017x – 0.046 and an *R*^2^ value of 0.9847).

The method described by Rahman et al. [53] utilized the aluminum chloride (AlCl_3_) colorimetric technique to assess the total flavonoid content (TFC) of either plant extracts under study or synthesized Ag-NPs, with slight modifications. A stock solution comprising AlCl_3_ (10%) and CH_3_CO_2_K (1M) was employed. The experiment was conducted in a 96-well microplate, with quercetin serving as the positive control. The spectral readings were measured at 430 nm using a microplate reader manufactured by Bio-Tek Instruments. For assessing TFC in either the plant extract or the Ag-NPs, a calibration curve was utilized and quantity of quercetin equivalent per gram of dry weight was used to calculate TFC. Quercetin concentrations in the standards varied from 25 to 100 µg mL^−1^, and the linear equation derived from the calibration curve was expressed as (y = 0.0323x – 0.2204, *R*^2^ of 0.9748).

#### Total Antioxidant Capacity (TAC) and Ferric Reducing Antioxidant Power (FRAP)

With a few slight modifications, the previously described phosphomolybdenum methodology was adopted to determine the sample’s total antioxidant capacity (TAC) by using phosphomolybdenum reagent [53]. The experiment was carried out in a 96-well microplate, and ascorbic acid was used as a positive control. The absorbance (Abs) was measured at 630 nm using a microplate reader (Bio-Tek Instruments) and calibration was carried out using ascorbic acid as a standard. The concentration of ascorbic acid in the standards varied from 25 to 100 µg mL^−1^, with a linear equation (y = 0.0323x—0.25, *R*^2^ 0.9784).

The FRAP of plant extract and synthesized Ag-NPs were measured using a previously reported method with slight modifications [53]. Positive controls included ascorbic acid, and samples were treated with phosphate buffer (1.15 mL, 0.20 M, pH 6.7) and potassium ferric cyanide (1.25 mL, 1.0%) at 20–100 µg/mL concentrations. FeCl_3_ (0.25 mL, 0.1%) and trichloroacetic acid (1.25 mL, 10%) were then added and mixed thoroughly using a vortex. Absorbance can be measured at 700 nm by spectrophotometer. Standard ascorbic acid was used for calibration. The concentration of ascorbic acid in the standards ranged from 25 to 100 µg mL^−1^, with a linear equation of (y = 201.47x – 32.12, *R*^2^ 0.9135).

#### DPPH (2,2-diphenyl-1-picrylhydrazyl) free radical scavenging assay

The antioxidant activity of the plant extract and Ag-NPs was measured by free radical, Diphenyl picryl hydrazyl (DPPH) scavenging activity, modified by Sharifi-Rad et al. [54] using a stock solution of DPPH. The experiment used a 96-well microplate with serial dilution (400, 200, 100, 50, and 25 µg/mL) and ascorbic acid was the positive control and DPPH radical working solution was the negative control. A Bio-Tek Instruments microplate reader recorded observations at 517 nm and calculated radical scavenging activity percentage (RSA%) using Eq. (1).

1 $$\mathrm{RSA}\;\%=\left[\left(\mathrm A\;_{\mathrm{control}}-\;{\mathrm A}_{\;\mathrm{samole}}\right)/\mathrm A\;_{\mathrm{control}}\right]\times100$$

Whereas A _control_ represents the absorbance of the control, while A _sample_ represents the absorbance of the tested sample.

#### Hydrogen peroxide (H_2_O_2_) free radical scavenging assay

Following a previously published methodology, the plant extract and synthesized Ag-NPs were tested for antioxidant activity by scavenging free radicals and H_2_O_2_ [55]. Hydrogen peroxide (H_2_O_2_) in phosphate buffer (50 mM, pH 7.4) was the solution of choice and after adding 0.1 mL of the test sample, 0.6 mL of H_2_O_2_ was added. Vortexing thoroughly mixing the mixture, and a spectrophotometer assessed the solutions’ absorbance at 230 nm after 10 min of incubation, using a blank sample and calculating H_2_O_2_ scavenging activity using Eq. (2).

2 $${\text{H}}_2{\text{O}}_2\,\text{scavenging activity}=\left[(1-{\text{A}}_\text{sample})/{\text{A}}_\text{control}\right]\times100$$

### Biocompatibility assay

#### Hemolytic assay

The hemolytic activity of the plant extract and synthesized Ag-NPs was evaluated using a protocol previously reported by Rodriguez et al. [56], with slight modifications. using red blood cells (RBCs). The supernatant from blood samples centrifuged at 4,000 rpm for 5 min was discarded and after three times phosphate-buffered saline (PBS) washes, the pellet was ready for erythrocyte suspension. The experiment was conducted in a 96-well microplate using serial dilutions (400, 200, 100, 50, and 25 µg/mL). The positive control was Triton X-100, and the negative control was DMSO. A Bio-Tek Instruments microplate reader monitored hemoglobin release at 540 nm and Eq. (3) was used to calculate hemolysis %.

3 $$\mathrm{Hermolysis}\;(\%)=\left[\left(\mathrm A\;_{\mathrm{control}}-\mathrm A\;_{\mathrm{sample}}\right)/\mathrm A\;_{\mathrm{conrol}}\right]\times100$$

#### Brine Shrimp Assay (BSA) for cytotoxic screening

The brine shrimp lethality assay was used with slight modification as described by Fatima et al. [57] using artificial sea water with a salinity of 35 g/L [58] and brine shrimps, and nanoparticles. The assay was performed in serial dilution (400, 200, 100, 50, and 25 µg/mL). The samples were introduced to the 96 well plates and the prepared sea water was added to the respective well contained the tested samples. For the experiment, DMSO was chosen as the negative control while doxorubicin was used as the positive control. After that, it was put in a suitable environment with an adequate amount of light and oxygen. After a day, the number of live and dead shrimp was counted using the following Eq. (5).

4 $$\mathrm{Lethality}\;\left(\%\right)=\left[\left({\mathrm{Nauplli}\;\mathrm{death}\;}_{\mathrm{before}\;\mathrm{treatment}}-{\mathrm{Nauplli}\;\mathrm{death}\;}_{\mathrm{after}\;\mathrm{treatment}}\right)/{\mathrm{Nauplli}\;\mathrm{death}\;}_{\mathrm{before}\;\mathrm{treatment}}\right]\times100$$

#### Phytotoxicity test for seed germination and root elongation

Plant extract and synthesized Ag-NPs were tested for phytotoxicity on seed germination and root elongation, following previously described methods with slight modifications [59, 60]. The *Raphanus sativus* seeds were surface sterilized in 3% H_2_O_2_ for 1 min, followed by 3–4 washing with deionized water. Radish seeds are commonly used in phytotoxicity tests. Subsequently, the seeds were placed in dH_2_O (control), plant extract, and Ag-NPs (100, 50, and 25 µg/mL), and shaken gently for 2 h. In 15 mm × 100 mm petri dishes, all seeds were placed on Whatman No.1 filter paper (90 mm diameter) and each filter paper had five radish seeds evenly dispersed. The petri dishes were filled with 5 ml of dH_2_O, plant extract, and Ag-NPs with different concentrations and incubated at 25 °C in darkness. After 3 d of incubation, the root length of each seedling was measured.

#### Antibacterial activity of Ag-NPs

The antibacterial activity of Ag-NPs was observed against pathogenic bacterial strains, employing the well diffusion method with slight modifications [61]. Various concentrations of Ag-NPs (100, 50, and 25 µg/mL) were tested against pathogenic bacteria including *B. subtilis*, *S. epidermidis*, *E. coli*, *P. aeruginosa*, *K. pneumonia*, and *S. typhi*. In 10 mL TSB, bacteria were grown for 24 h at 37 °C with 120 rpm stirring and a standardized inoculum (1 × 108 CFU/mL) was prepared for each bacterial strain. DMSO served as the negative control. All TSB-cultured bacteria incubate at 37 °C for 24 h while shaking. Next, 50 L of grown bacterial cell suspensions were spread on Tryptic soy agar (TSA) media petri plates using an autoclave cotton swab, followed by the formation of 10 mm wells at a specific distance from each other. Different Ag-NPs concentrations were added to the well. along with positive control ampicillin and incubated at 37 °C for 24 h. The bacterial strains’ inhibitory zones were measured in mm by vernier calliper after 24 h.

#### Statistical analysis

GraphPad Prism 9 and OriginPro 9.0 were used for statistical analysis, using ANOVA and Tukey’s HSD test for multiple comparisons at a 95% significance level (*p* < 0.05). Results from triplicate trials are reported as mean ± standard error (SE).

## Results

### Physicochemical characterization of synthesized Ag-NPs

UV–Vis, XRD, FT-IR, Raman, SEM–EDS, Zeta potential determination, DLS, TGA, and DSC were used to confirm the synthesis of silver nanoparticles.

### UV–Vis absorption spectrophotometry and X-ray diffraction (XRD)

Figure 1-a depicts the UV–Vis spectrum of synthesized Ag-NPs using leaf extract from *W*. *coagulans*. This spectrum illustrates the eco-friendly production of Ag-NPs through the reaction between silver nitrate solution and *W*. *coagulans* leaf extract. The change in color from yellow to dark brown indicates the successful formation of Ag-NPs. Furthermore, the observation of a clear peak at 420 nm provides evidence of the efficient synthesis and stability of Ag-NPs. In contrast, *W*. *coagulans* leaf extract exhibits a peak at 307 nm, while the silver salt solution shows a peak at 207 nm.

Figure 1-b shows the X-ray diffraction (XRD) pattern of Ag-NPs, which was produced using an aqueous extract of *W. coagulans*. Based on the observed peaks at 2 theta (θ) angles of 38.14°, 44.36°, 77.98°, and 82.03°, it can be confirmed that the facets corresponding to these angles are (100), (002), (101), and (002), respectively. It confirms the face-centered cubic (fcc) structure of Ag-NPs. These facets correspond to the data in the Joint Committee on Powder Diffraction Standards (JCPDS), file No. 01–073–1667. The average Ag-NP size was calculated using the Debye–Scherrer formula (Eq. 5; Table 1). Table 1 shows the average Ag-NP size was 39.76 nm.

5 $$D=\frac{0.94\lambda}{\beta\;COS\theta}$$

**Table 1:** The crystallite size of green synthesized Ag-NPs

XRD Analysis of Ag-NPs by Debye–Scherrer equations												
**Peak No**	**2θ**	**Θ**	**θ (Radians)**	**cos(θ)**	**Sin(θ)**	**sin^2(θ)**	**(hkl)**	d = λ/(2*Sinθ)	a = d*(h^2 + k^2 + l^2)^1/2	**β**	**β (Radians)**	D = K*λ/(βcosθ)
1	38.14	19.07	0.332	0.945	0.326	0.106	(100)	0.235	0.117	0.16	0.002	52.51
2	44.36	22.18	0.387	0.926	0.377	0.142	(002)	0.203	0.407	0.33	0.005	25.98
3	77.98	38.99	0.680	0.777	0.629	0.395	(101)	0.122	0.122	0.21	0.003	48.65
4	82.03	41.01	0.715	0.754	0.656	0.430	(002)	0.117	0.234	0.33	0.005	31.89

Crystallite particle size = 39.76 nm

### Fourier transform infrared spectroscopy (FT-IR) and Raman spectra

**Fig. 3:**
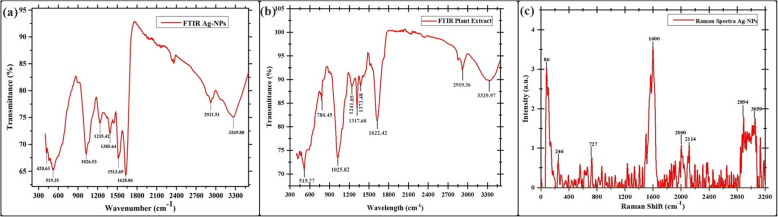
Physicochemical characterizations of biosynthesized Ag-NPs. **a** FTIR spectra of Ag-NPs, **b** FTIR spectra of *W*. *coagulans*, **c** Raman spectra of Ag-NPs

The FT-IR analysis provides valuable insights into the molecular composition and structure of the sample. The FT-IR spectrum obtained for the plant extract reveals several prominent peaks, each corresponding to specific functional groups and vibrational modes of chemical bonds. The analysis of silver nanoparticles using *W*. *coagulans* extract via FT-IR spectroscopy revealed notable bands and diverse functional groups as shown in Fig. 3a, including 3269.88 cm^−1^ (intense O–H stretching characteristic of alcohol), 2921.51 cm^−1^ (vigorous O–H stretching typical of carboxylic acid), 1628.86 cm^−1^ (moderate O–H stretching indicative of alkene), 1513.69 cm^−1^ (vigorous N–O stretching suggesting a nitro compound), 1385.64 cm^−1^ (moderate C = C stretching associated with alkane), 1235.42 cm^−1^ (vigorous C-O stretching attributed to alkyl aryl ether), 1026.53 cm^−1^ (vigorous C-N stretching indicating amine presence), 519.35 cm^−1^ (vigorous C–Br stretching of halo compound), 420.63 cm^−1^ (moderate C–Br bending of alkyl halide compound). The FT-IR spectrum of the plant extract suggests a complex mixture of organic compounds (Fig. 3b). The presence of broad O–H and N–H stretching peaks around 3329.97 cm^−1^ indicates the presence of alcohols, phenols, and possibly amines, suggesting a high degree of hydrogen bonding. The strong C-H stretching peak at 2919.36 cm^−1^ points to aliphatic hydrocarbons, likely indicating the presence of long-chain alkanes or fatty acids. The peak at 1622.42 cm^−1^ reveals the presence of unsaturated compounds such as alkenes or aromatic compounds, along with potential amine or amide functionalities. Finally, the C-O stretching peak at 1025.82 cm^−1^ highlights the presence of compounds like alcohols, ethers, or esters. The 1373.48 cm^−1^ peak is associated with C-H bending vibrations, suggesting methyl groups or secondary alcohols. The 1317.68 cm^−1^ peak corresponds to C-H bending in aromatic compounds or O–H bending in alcohols and phenols, indicating aromatic rings or phenolic structures. The 1241.85 cm^−1^ peak is characteristic of C-O stretching vibrations, pointing to ethers, esters, or phenolic groups. The 780.45 cm^−1^ peak indicates out-of-plane C-H bending in aromatic compounds and alkenes, suggesting the presence of aromatic rings or unsaturated hydrocarbons. The 515.77 cm^−1^ peak is associated with C-X stretching in halogenated compounds or S–S stretching in sulfides, indicating possible halogenated compounds or sulfide linkages.

The Raman spectrum of silver nanoparticles contains several significant bands that correspond to different functional groups or vibrational modes and is used to further characterize the structural changes in the Ag-NPs. The Raman spectrum of Ag-NPs shows several prominent peaks at specific Raman shifts, each indicating different vibrational modes and functional groups. Figure 3c shows that the peaks at 86 cm^−1^ and 246 cm^−1^ are associated with lattice vibrations of the silver nanoparticles. A peak at 727 cm^−1^ suggests the presence of adsorbed molecules, potentially related to C-H out-of-plane bending modes. The intense peak at 1600 cm^−1^ indicates strong C = C stretching vibrations in aromatic compounds, highlighting significant Raman activity likely enhanced by the surface-enhanced Raman scattering (SERS) effect of the nanoparticles. Peaks at 2000 cm^−1^ and 2114 cm^−1^ may correspond to combination bands or specific molecular interactions, such as nitrile (C≡N) stretching. The peaks at 2894 cm^−1^ and 3059 cm^−1^ are indicative of C-H stretching vibrations, with 2894 cm^−1^ corresponding to aliphatic hydrocarbons and 3059 cm^−1^ to aromatic C-H stretches. Together, these peaks provide insights into the structural and chemical properties of the silver nanoparticles and their surface interactions.

### SEM–EDS, HR-TEM examination, and particles size distribution

**Fig. 4:**
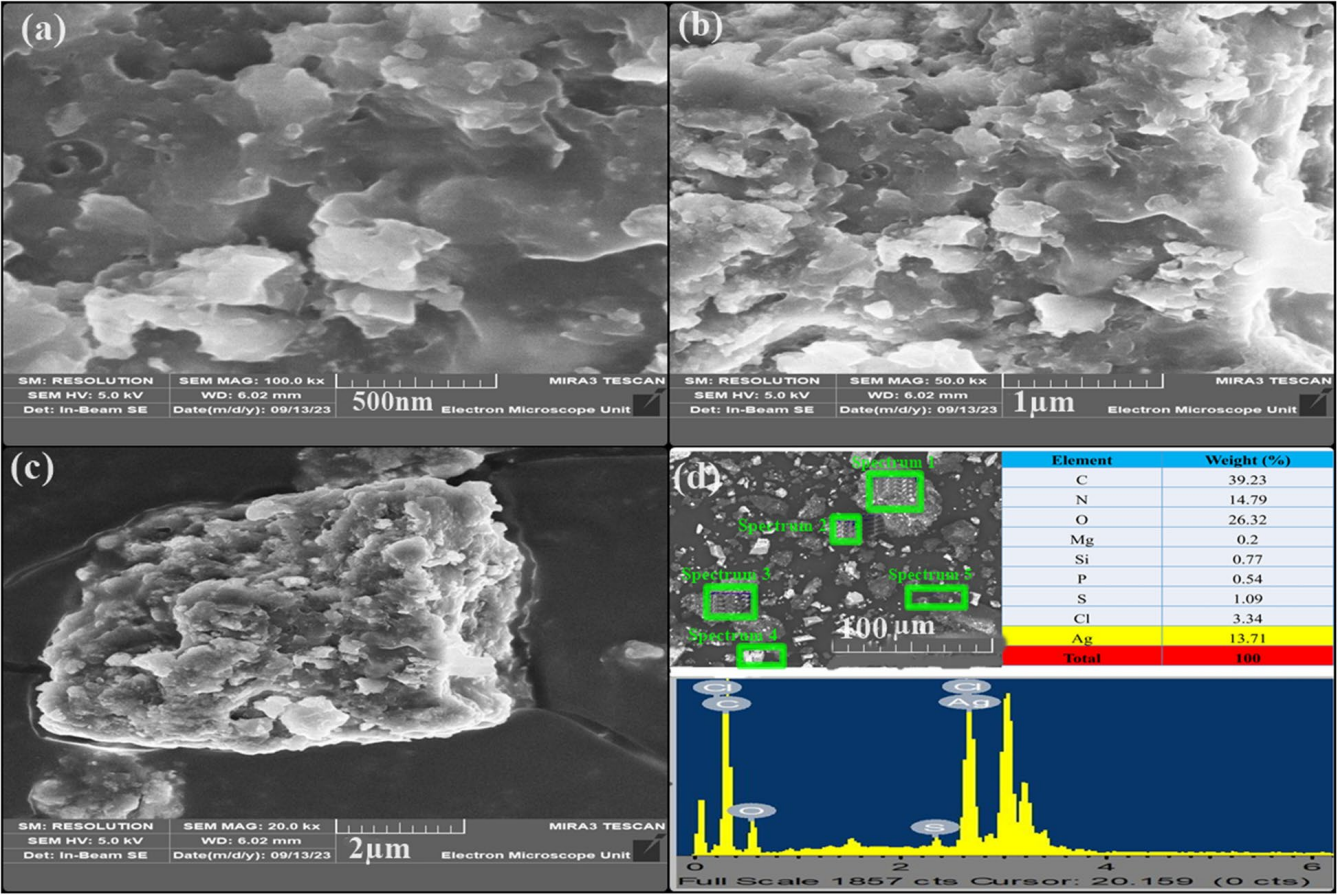
**a** The SEM image at various magnifications (**a-c**) and (**d)** the energy dispersive spectroscopy (EDS) of the Ag-NPs

**Fig. 5:**
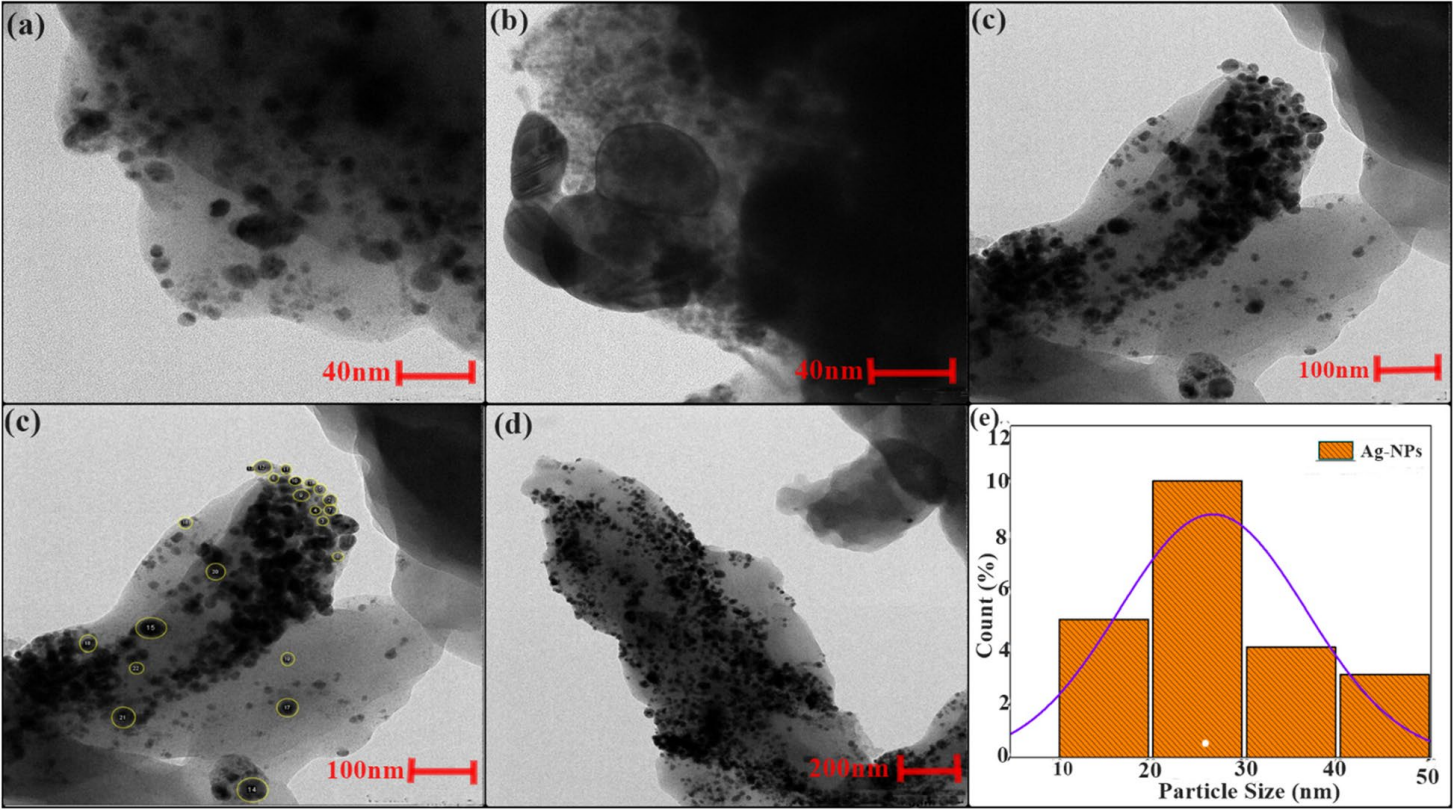
HR-TEM images of Ag-NPs at various magnifications (**a-d**) and **e** Particle distribution size of the Ag-NPs

Using Scanning Electron Microscopy (SEM) with Energy Dispersive Spectroscopy (EDS) analysis, the surface nature and elemental configuration of the Ag-NPs were determined (Fig. 4a-d). The SEM and HR-TEM results indicate that the Ag-NPs exhibit an irregular shape, with the HR-TEM revealing a particle diameter of approximately 26.63 nm, as depicted in Figs. 4 and 5. The EDS analysis was performed to evaluate the elemental composition of the Ag-NPs. Using the EDS spectrum, the weight percentage of the Ag-NPs was determined, with 13.71 keV observed in Fig. 4d.

### Characterizing zeta potential and DLS

**Fig. 6:**
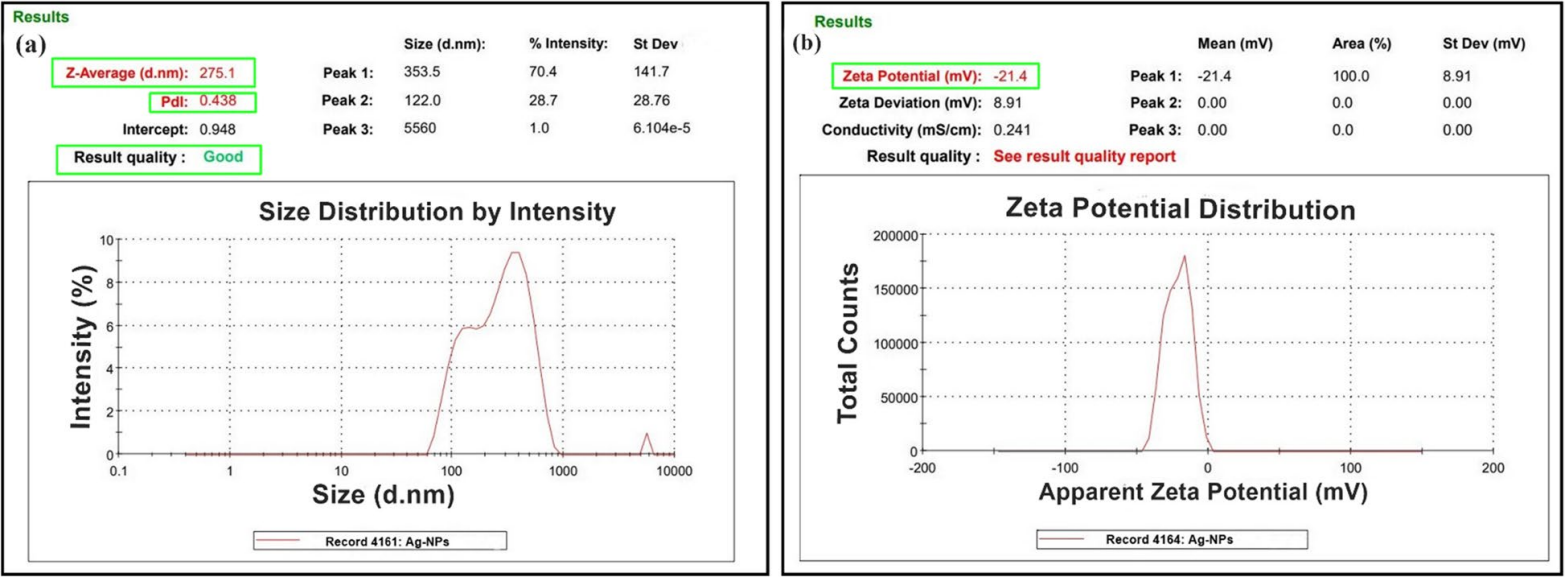
Zeta Potential and DLS analysis of Ag-NPs using the *W. coagulans* extract showing. **a** the size distribution by intensity, **b** Zeta potential distribution

Malvern Instruments Zeta sizer, version 8.00.4813, used dynamic light scattering to assess the silver nanoparticle solution’s intensity-based particle size distribution and zeta potential. Dynamic light scattering measures the thickness of the capping or stabilizing material surrounding metallic particles and the average size distribution of green synthesized Ag-NPs in solution. Figure 6a shows the 275.1 nm size and 0.438 polydispersity index (PDI). The fluorescent green Ag-NPs had a zeta potential of − 21.4mV, as shown in Fig. 6b. The Ag-NPs PDI value is < 0.7, showing excellent silver nanoparticle fabrication utilizing *W. coagulans* extract. The nanoparticles PDI of 0.438 indicates their well-defined dimensions and strong monodispersity.

### TGA–DSC analysis of Ag-NPs

**Fig. 7:**
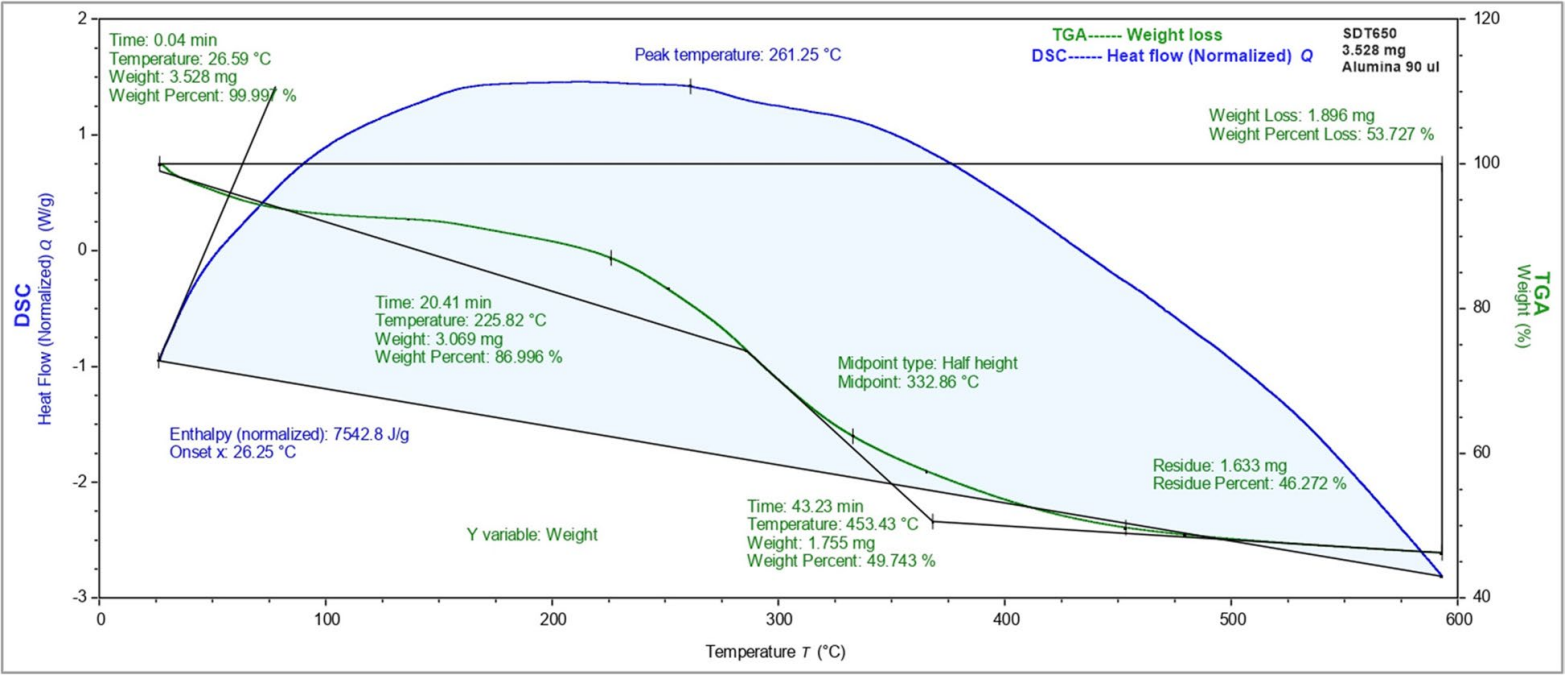
TGA–DSC curves of biosynthesized Ag-NPs in N_2_ atmosphere (heating rate: 10 °C min^−1^, flow rate: 50 mL min.^−1^)

The thermal properties of the manufactured material were additionally explored through DSC-TGA characterization using a TA instrument. DSC and TGA curves from a prepared Ag-NPs powder sample are shown in Fig. 7. In the TGA analysis, three distinct weight loss stages were identified. The initial weight loss of 0.01% occurred at 26.59 ºC, followed by a substantial loss of 13% at 225.82 ºC, and a further significant loss of 37.25% at 453.43 ºC. Additionally, 3.47% residue weight loss was observed in the first, second, and third regions, respectively. The total weight of Ag-NPs reduced from 3.528 mg to 1.896 mg, indicating an overall 53.727% weight loss as depicted in Fig. 7. The initial 0.01% weight loss during the heating phase from 25 to 187 °C is attributed to the evaporation of water molecules from the outer surface of Ag-NPs. The subsequent 13% weight loss during the range of 188 to 366 ºC is associated with the degradation and evaporation of organic molecules acting as stabilizing agents on the surface of Ag-NPs.

### Phytochemical analysis and antioxidant activities of Ag-NPs TPC, TFC, TAC and FRAP activities of Ag-NPs

**Fig. 8:**
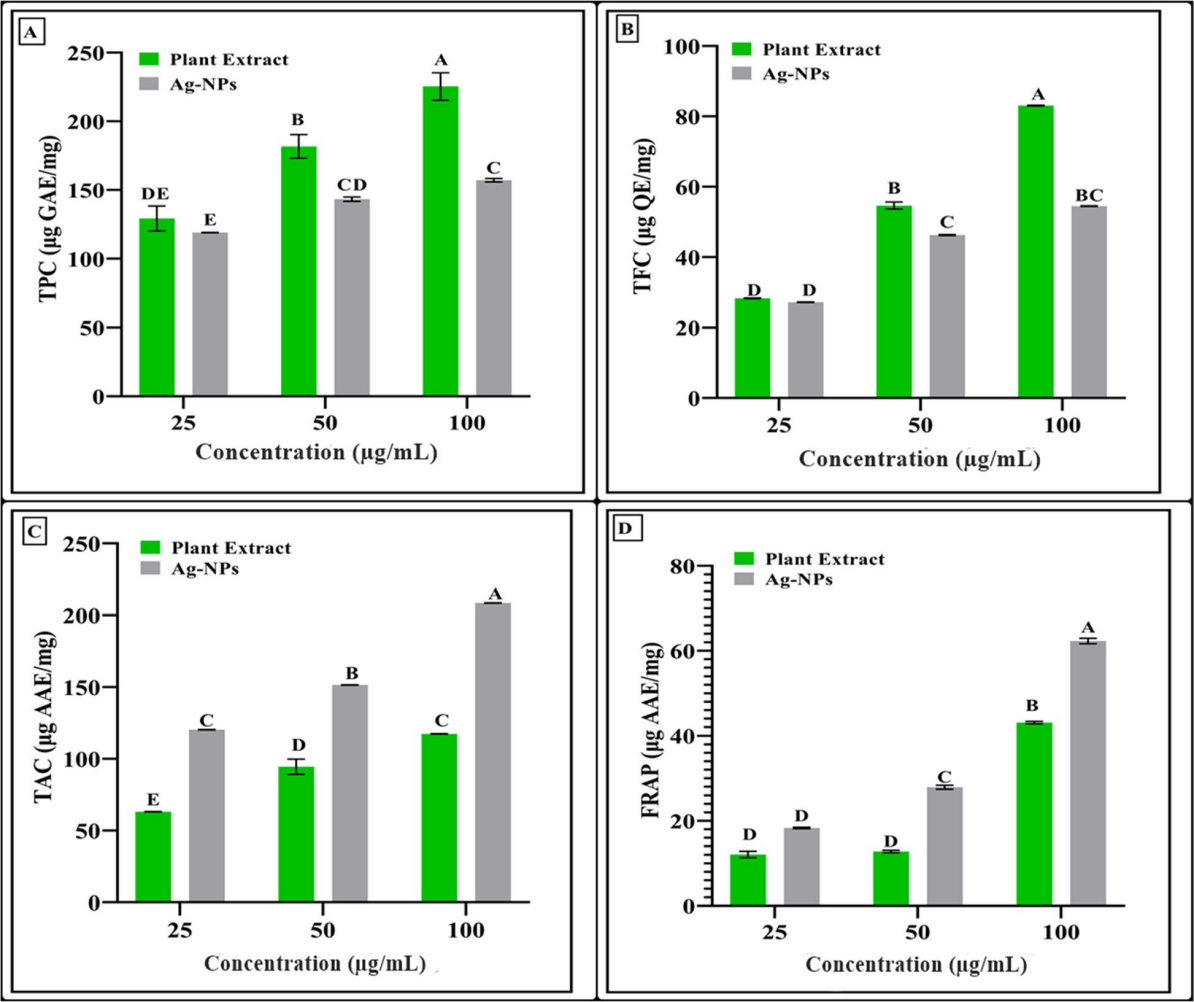
Phytochemical and antioxidant analysis of Ag-NPs using the *W. coagulans* extract showing; **a** Total phenolic contents (TPC), **b** Total flavonoids content (TFC), **c** Total antioxidant capacity (TAC) and **d** Ferric reducing antioxidant power (FRAP). Error bars show the standard error meaning of three replicates, and letters indicate Tukey’s HSD-based significant differences between tested concentrations at (*p* < 0.05)

**Fig. 9:**
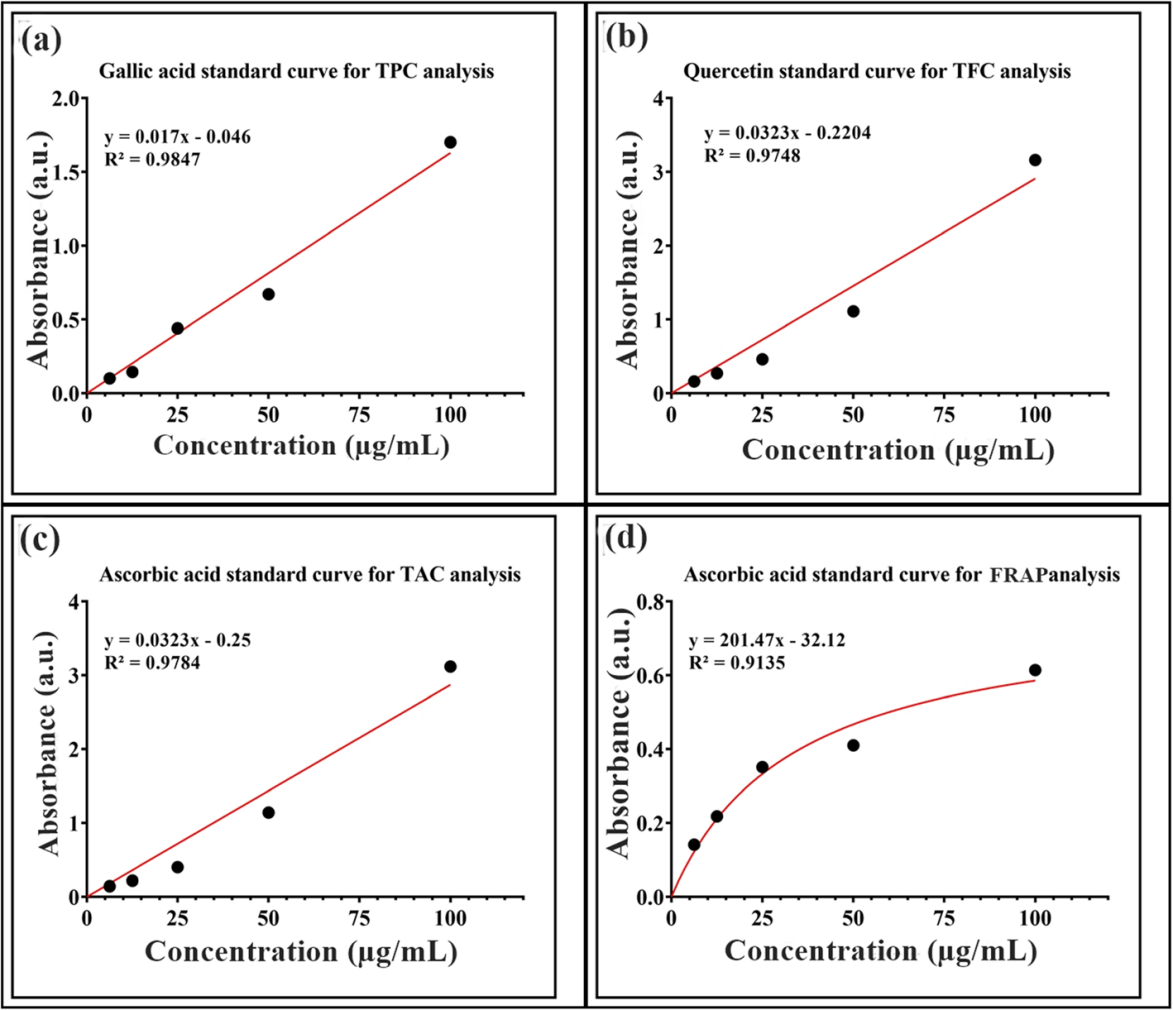
Standard curve of phytochemical and antioxidant analysis of Ag-NPs using the *W. coagulans* extract showing; **a** Total phenolic contents (TPC), **b** Total flavonoids content (TFC), **c** Total antioxidant capacity (TAC) and **d** Ferric reducing antioxidant power (FRAP)

The synthesized Ag-NPs and leaf extract of *W. coagulans* shows the presence of a wide range of TPC, TFC, TAC and FRAP as shown in Figs. 8a-d & 9a-d (standard curve). Both the leaf extract and Ag-NPs demonstrated a dose-dependent increase in the TPC and TFC. Higher concentrations of Ag-NPs led to elevated TPC values, with those synthesized through a green method ranging from 119.06 to 157.13 g GAE/mg dry weight, while *W*. *coagulans* leaf extract exhibited values ranging from 129.33 to 225.36 g GAE/mg dry weight, respectively. Similarly, higher concentrations of Ag-NPs resulted in higher TFC values, ranging from 27.18 to 54.53 g QE/mg dry weight, whereas the *W*. *coagulans* leaf extract showed values ranging from 28.30 to 83.07 g QE/mg dry weight, respectively. As shown in Fig. 8a, the Ag-NPs showed higher values of total antioxidant capacity (TAC) as compared to plant extract which ranged from 120.23 to 208.64 g AAE/mg dry weight and 63.15 to 117.44 g AAE/mg dry weight, respectively. As depicted in Fig. 8a, Ag-NPs demonstrated a notably higher capability for converting Fe^3+^ to Fe^2+^ in the FRAP assay when compared to the *W. coagulans* leaf extract. The differences were observed in a range of concentrations from 25 to 100 g/mL, with values that range from 18.30 to 62.28 g AAE/mg dry weight for Ag-NPs.

### DPPH and hydrogen peroxide (H_2_O_2_) free radical scavenging assay

**Fig. 10:**
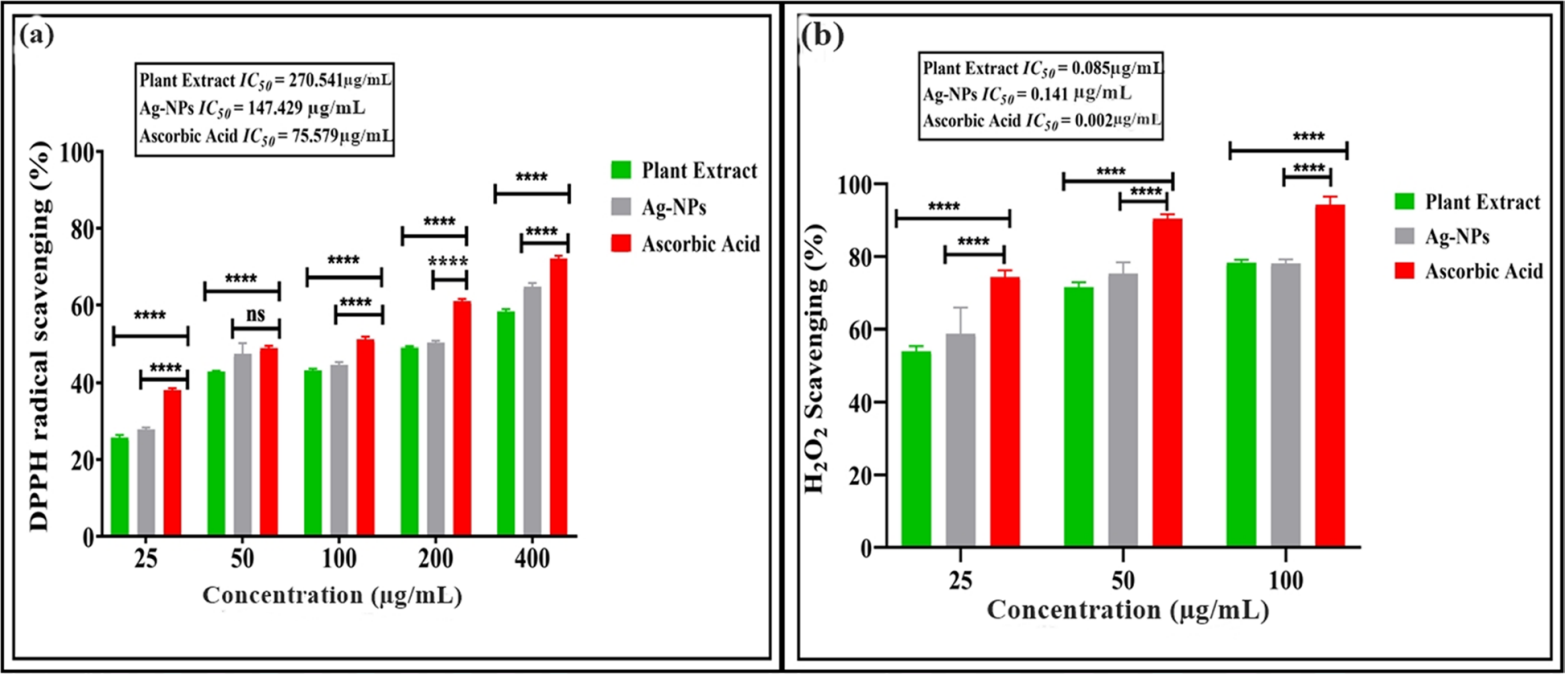
**a** The 2,2-diphenyl-1-picrylhydrazyl (DPPH) radical scavenging activity, **b** Hydrogen peroxide (H_2_O_2_) free radical scavenging activity of the *W. coagulans* leaf extract and Ag-NPs. Error bars show the standard error meaning three replicates; asterisks indicate significant differences (*p* < 0.05)

The antioxidant activity of synthesized Ag-NPs and aqueous *W. coagulans* leaf extract was evaluated using DPPH free radical and H_2_O_2_ assays (Fig. 10a-b). DPPH, a stable molecule reduced by hydrogen or electrons, is commonly used to assess antioxidant potential. Lower *IC*_*50*_ values indicate stronger DPPH scavenging activity by the extract, while higher values suggest lower activity. Figure 10a illustrates DPPH radical antioxidant activity at various Ag-NP concentrations. Our findings demonstrate that both aqueous *W. coagulans* leaf extract and synthesized Ag-NPs exhibit free radical scavenging activities. However, Ag-NPs exhibit more effective DPPH scavenging compared to the *W. coagulans* extract. The DPPH and H_2_O_2_ activity of Ag-NPs and *W. coagulans* extract increased dose-dependently. Ag-NPs DPPH scavenging activity ranged from 27.89% to 64.85% at concentrations of 25–400 g/mL, with an average *IC*_*50*_ value of 147.429 (Fig. 10a). In comparison to Ag-NPs, the aqueous leaf extract shows a higher level of potency in scavenging H_2_O_2_, the H_2_O_2_ scavenging activity ranged from 54.11% to 78.25% at concentrations of 25–100 g/mL, with an average *IC*_*50*_ value of 0.085 (Fig. 10b).

### Hemolysis and Brine Shrimp Assay (BSA) for cytotoxicity screening

**Fig. 11:**
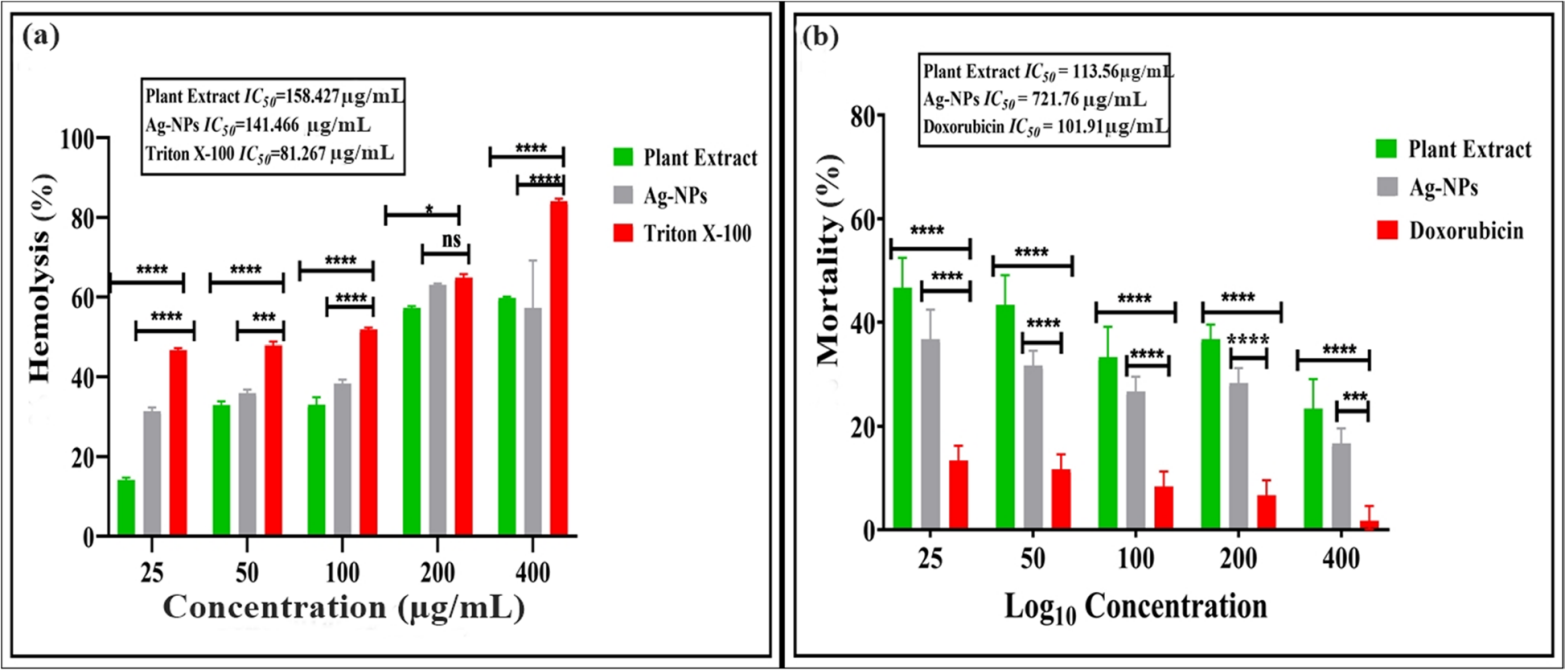
**a** Hemolytic activity in human red blood cells, **b** In vitro cytotoxic activity of biosynthesized Ag-NPs in Brine shrimp lethality test of the *W. coagulans* leaf extract and Ag-NPs. Error bars show the standard error meaning of three replicates; asterisks indicate significant differences (*p* < 0.05)

The toxicity assessment of synthesized Ag-NPs and aqueous *W. coagulans* leaf extract were evaluated using % hemolysis and Brine Shrimp Assay (BSA) (Fig. 11a-b). Figure 11a illustrates % hemolysis and brine shrimp lethality cytotoxicity activities at various concentrations of Ag-NPs and *W. coagulans* leaf extract. Our findings show that both aqueous *W. coagulans* leaf extract and synthesized Ag-NPs exhibit cytotoxicity activities. However, Ag-NPs exhibit more effective % hemolysis compared to the *W. coagulans* extract. The % hemolysis and BSA activity of Ag-NPs and leaf extract increased dose-dependently. In comparison to aqueous leaf extract, Ag-NPs shows a higher level of potency in % hemolysis ranged from 31.42% to 63.02% at concentrations of 25–400 g/mL, with an average *IC*_*50*_ value of 141.466. However, the aqueous leaf extract shows % hemolysis ranged from 14.14% to 59.79% at concentrations of 25–400 g/mL, with an average *IC*_*50*_ value of 158.427. Triton X-100 the positive control showed an average *IC*_*50*_ value 81.267 g/mL at 25–400 g/mL.

The brine shrimp cytotoxicity assay proves to be an effective approach for conducting preliminary in vitro toxicity assessments [62]. Nauplii were exposed to varying concentrations (25–400 g/mL) of plant extract and Ag-NPs for a duration of 24 h. The potential of the plant extract and Ag-NPs were assessed by counting the number of motile nauplii. In comparison to Ag-NPs, aqueous leaf extract showed high lethality activity against brine shrimp larvae with an average 113.56 g/mL *IC*_*50*_ value at concentrations of 25–400 g/mL and Ag-NPs have an average 721.76 g/mL *IC*_*50*_ value at concentrations of 25–400 g/mL (Fig. 11-b). Doxorubicin the positive control showed an average *IC*_*50*_ value 101.91 g/mL at 25–400 g/mL.

### Phytotoxicity test of green synthesized Ag-NPs

**Fig. 12:**
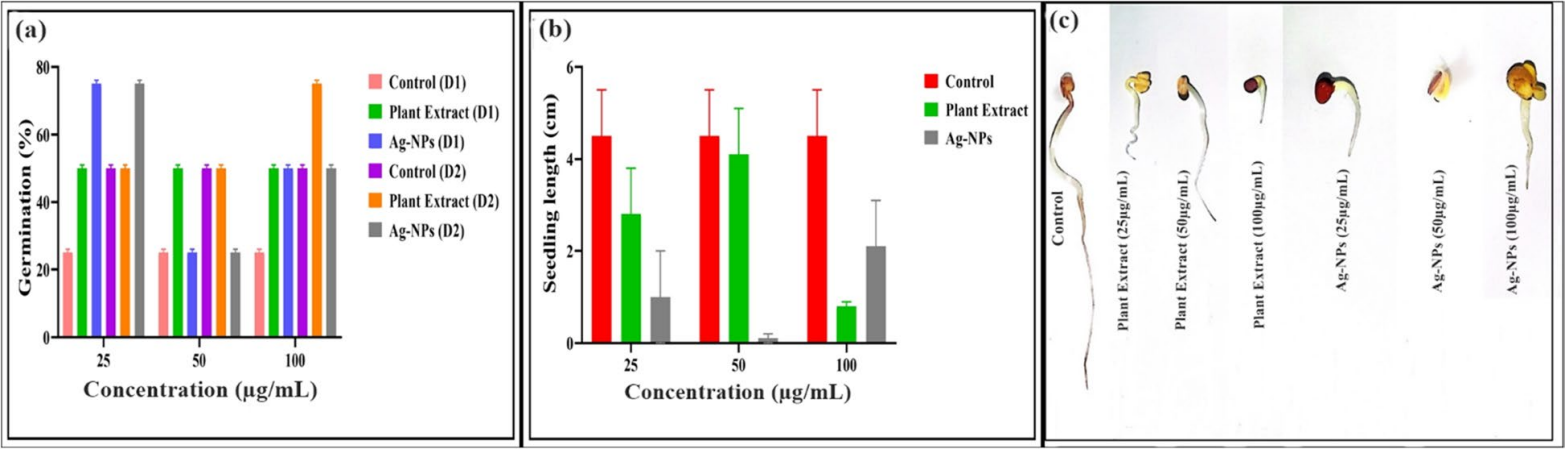
**a** Phytotoxicity effect of Ag-NPs and *W. coagulans* leaf extract on germination percentage of radish seeds, **b**, **c** seedling length. Error bars represent the standard error of three replicates

In this study, various concentrations (25, 50, and 100 µg/mL) were initially chosen to explore the phytotoxicity of the selected nanoparticles and *W. coagulans* leaf extract on radish seeds as shown in Fig. 12a-c. Following the USEPA guidelines, it is imperative to ensure that the nanoparticles do not induce adverse effects on seed germination and root growth, even at elevated concentrations, to alleviate toxicity in test plants [63]. It’s important to note the impact of nanoparticles at various concentrations (25, 50, and 100 µg/mL) on seed germination and seedling length, as illustrated in Fig. 12a-c. Both types of Ag-NPs and plant extract showed higher germination percentages on day 1 and day 2 compared to the control at different concentrations, ranging from 25–75%. Conversely, *W. coagulans* leaf extract exhibited percentages ranging from 50–75% compared to the control (distilled water). The influence of nanoparticles on seedling growth varied based on the concentration and the type of nanoparticles and plant extract used. It’s worth noting the high level of phytotoxicity observed when using Ag-NPs and plant extract. Their suspensions significantly affected the length of radish seedlings, with seedling length reduced by 38%, 9%, and 82% using *W. coagulans* leaf extract and Ag-NPs, and by 78%, 97%, and 53% at 25, 50, and 100 µg/mL, respectively, compared to the control (distilled water). Our study findings suggest that radish seeds treated with Ag-NPs for 2 days in the phytotoxicity assay exhibited an increased germination rate alongside a decrease in seedling length.

### Antibacterial activities of green synthesized Ag-NPs

**Fig. 13:**
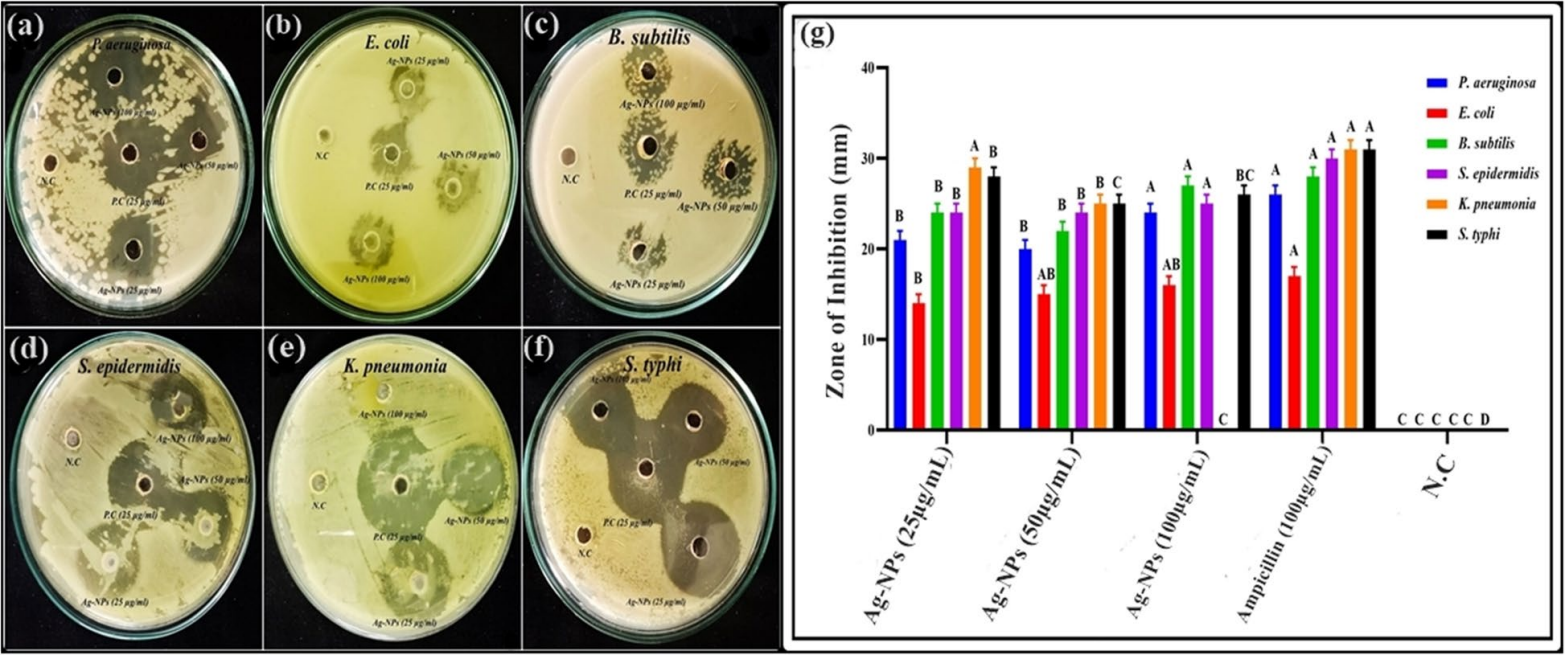
Antibacterial activities of Ag-NPs; **a** *P. aeruginosa,* **b** *E. coli,* **c** *B. subtilis*, **d** *S. epidermidis* **e** *K. pneumonia* **f** *S. typhi*, **g** Antibacterial activities of Ag-NPs against different pathogenic bacterial strains. Error bars show the standard error mean of three replicates, and letters indicate Tukey’s HSD-based significant differences between tested concentrations at (*p* < 0.05)

In the study, the potential of silver nanoparticles at different concentrations (25, 50, 100 g/mL) in combatting diverse pathogenic bacterial strains, including *Bacillus subtilis*, *Staphylococcus epidermidis*, *Escherichia coli*, *Pseudomonas aeruginosa*, *Klebsiella pneumonia*, and *Salmonella typhi* (Fig. 13a-g). The findings indicate that Ag-NPs exhibited higher antibacterial activity against a range of pathogenic bacteria as compared to negative control tryptic soy broth (TSB), as depicted in Fig. 13a-g. By employing different concentrations of Ag-NPs synthesized through green methods (25, 50, 100 g/mL), the antibacterial activity was assessed utilizing the well diffusion technique, and the zones of inhibition (ZOI) of bacteria were observed. In comparison to other bacterial strains, *K. pneumonia* and *S. typhi* exhibited the highest zone of inhibitory activity at 25 L, measuring 29 ± 0.01 mm, and 28 ± 1.00 mm while *E. coli* displayed the lowest zone of inhibitory activity at 25 L, measuring 14 ± 1.01 mm (Fig. 13g). No detection of ZOI was observed in the TSB media under negative control conditions. Compared to the control, the recorded values show statistical significance (*p* < 0.05). The results showed that inhibition diameters for different bacteria ranged from 14 to 29 mm.

## Discussion

The use of *W. coagulans* leaf extract to produce silver nanoparticles has shown a sustainable and environmentally friendly method for synthesizing nanoparticles. This method exploits the reducing and stabilizing properties of plant phytochemicals, reducing the need for toxic chemicals commonly used in conventional nanoparticle production. In the field of green synthesis of Ag-NPs, various plant extracts have been employed as reducing and stabilizing agents, with *W. coagulans* showing significant potential. Previously, Qasim et al. [64] reported the successful synthesis of iron oxide nanorods using *W. coagulans* extract, underscoring its effectiveness in nanoparticle formation. Similarly, *Moringa oleifera* (drumstick tree) has been widely studied, with Aisida et al. [65] and Mehwish et al. [66] noting its effective reducing and stabilizing properties in AgNP synthesis, accompanied by antimicrobial and photocatalytic activities. *Azadirachta indica* (neem) is another frequently used source; Tripathy et al. [9] highlighted its role in biomimetic AgNP synthesis, while Velusamy et al. [67] demonstrated its potential for producing stable, antibacterial silver nanoparticles. *Ocimum sanctum* (holy basil) has also been employed in AgNP synthesis, with Ramteke et al. [68] observing enhanced antibacterial activity, and Malapermal et al. [69] reporting additional antidiabetic and antioxidant properties. In comparison, *W. coagulans* offers similar reducing and stabilizing capabilities to these other plant extracts, yet provides advantages in yielding uniform, stable nanoparticles with consistent size and morphology due to its balanced phytochemical profile, rich in flavonoids and phenolic compounds. While other extracts like *A. indica*, *M. oleifera*, and *O. sanctum* are effective, they often produce broader particle size distributions, indicating less control over stabilization. The unique properties of *W. coagulans* make it a promising bioreductant for green synthesis, with stable, well-defined Ag-NPs suitable for phytochemical, antioxidant, cytotoxic and biomedical applications, as evidenced by the findings of this study.

The synthesized Ag-NPs were characterized using various techniques. UV–Vis spectroscopy confirmed the formation of Ag-NPs by exhibiting a distinct surface plasmon resonance (SPR) peak at about 420 nm, consistent with previous studies [70]. The change in color occurs due to the optical properties of Ag-NPs and is known as localized surface plasmon resonance (SPR) [71]. Fotoohiyan et al. [72] observed a peak in the UV–vis spectrum of the Ag-NPs synthesized at 420 nm, as documented in their study. Furthermore, Li et al. [73] noted a similar peak absorbance at 420 nm for their Ag-NPs. The results align with the findings of our investigation. The size of these silver nanoparticles correlates with earlier research on green synthesized nanoparticles [74, 75]. X-ray diffraction (XRD) analysis confirmed the crystalline nature of the Ag-NPs, with diffraction peaks at 38.14°, 44.36°, 77.98°, and 82.03° corresponding to Ag-NPs crystallite planes (100), (002), (101), and (002), aligning with Rafique et al. [76] corresponding to the face-centered cubic structure of silver. Fourier-transform infrared spectroscopy (FT-IR) analysis revealed the presence of biomolecules from the *Withania coagulans* leaf extract on the surface of the Ag-NPs, highlighting their role in reduction and stabilization. The observed functional groups act as capping agents, coordinating with silver atoms on the nanoparticle surface to prevent aggregation, thereby controlling particle size and shape, as supported by previous studies [77]. Specifically, the broad O–H stretching bands at 3269.88 cm^−1^ and 2921.51 cm^−1^ indicate the involvement of hydroxyl and carboxyl groups, which likely facilitate the reduction of Ag^+^ to Ag^0^. The C = C stretching vibrations at 1600 cm^−1^ suggest the presence of aromatic compounds, while peaks at 2000 cm^−1^ and 2114 cm^−1^ may indicate nitrile (C≡N) stretching or other molecular interactions contributing to nanoparticle stability. Further peaks at 2894 cm^−1^ and 3059 cm^−1^ correspond to C-H stretching vibrations, with 2894 cm^−1^ associated with aliphatic hydrocarbons and 3059 cm^−1^ with aromatic C-H stretches. These findings suggest that the diverse functional groups in the *W. coagulans* extract, including hydroxyl, carboxyl, and aromatic groups, interact with silver ions, acting as both reducing and stabilizing agents. This coordination not only aids in the reduction process but also stabilizes the nanoparticles by capping their surfaces, preventing aggregation, and maintaining a controlled particle size and shape. These results align well with the findings of our study, reinforcing the critical role of these biomolecules in the biosynthesis of Ag-NPs. Together, these peaks provide insights into the structural and chemical properties of the silver nanoparticles and their surface interactions this Raman shift aligns with the results documented in earlier studies [78]. In TGA–DSC the remarkable stability in weight throughout these processes suggests that the synthesized nanoparticles exhibit excellent thermal stability, findings align closely with the previous studies as reported by Alshammari et al. [79]. According to Younas et al. [80], the final and steady 37.25% weight loss from 366 to 800 °C is because more stable organic molecules that are bound to the surface of Ag-NPs dissociate out. Nevertheless, the alignment between the DSC spectra and TGA results occurs as there is a rise in heat stemming from exothermic reactions triggered by the dissociation of evaporated molecules [81]. Transmission electron microscopy (TEM) revealed that the Ag-NPs were predominantly irregular with a size distribution ranging from 35 to 40 nm. The current results of SEM analysis align with the findings of previous research [82] and our results of EDS analysis strongly corelates with the previous studies [83]. The results of our Zeta Potential and DLS findings align closely with the previous studies [84]. The initial, final, and steady weight loss of Ag-NPs can be attributed to the evaporation of water molecules, degradation and evaporation of organic molecules, dissociation of organic molecules that are more stable and bound to the Ag-NPs surface. Nevertheless, the alignment between the DSC spectra and TGA results occurs as there is a rise in heat stemming from exothermic reactions triggered by the dissociation of evaporated molecules [79–81]. Compared to other plant-extracts-synthesized Ag-NPs, *W. coagulans* Ag-NPs exhibit unique advantages, including a stable, controlled particle size distribution (35–40 nm) and exceptional thermal stability, as evidenced by TGA–DSC analysis. These properties, combined with the effective capping and reducing action of *W. coagulans* phytochemical profile, indicate superior nanoparticle stability and size uniformity. Such characteristics make *W. coagulans* Ag-NPs particularly promising for applications that require high stability, such as in biomedical and environmental fields.

Studies have shown that plant extracts can serve as stabilizing agents for Ag-NPs, preventing aggregation and enhancing their uptake by plants. However, these interactions can also amplify phytotoxic effects because the presence of plant extract components may facilitate the release of silver ions from Ag-NPs, increasing their toxicity to plants. The results align with prior research findings, indicating consistency in the phytotoxic effects observed from the combination of plant extracts and silver nanoparticles [85]. The bioactivity assays revealed that the synthesized Ag-NPs exhibit significant antibacterial and antioxidant activities. Antimicrobial activity has been evaluated against variety of bacterial strains, including both Gram-positive and Gram-negative bacteria. The Ag-NPs had an extensive antibacterial impact, which can be attributed to the disruption of microbial cell membranes and the production of reactive oxygen species (ROS). The antioxidant activity of the silver nanoparticles was evaluated using the DPPH radical scavenging assay. The results showed the scavenging activity of the nanoparticles increased in a dose-dependent manner. The antioxidant potential of the *W. coagulans* leaf extract could be attributed to the presence of phenolic compounds, which are known for their redox properties. Phenolic compounds found in phytochemical screening were observed to have an impact on the biological activity and functionality of Ag-NPs. These findings are in line with previous research that suggests these compounds are responsible for reducing Ag(I) ions and stabilizing Ag-NPs [86]. Our results closely align with previous studies, as highlighted by Sharifi-Rad et al. [54]. In line with the research conducted by Salari et al. [87], the synthesized Ag-NPs revealed higher TAC and FRAP values compared to the leaf extracts of *W. coagulans*. Lower *IC*_*50*_ values indicate stronger free radical scavenging activity and results of our DPPH and H_2_O_2_ findings align closely with the previous studies [87]. The % hemolysis and brine shrimp lethality cytotoxicity activities results of our findings corelates with the previous studies as the concentration increases, the lethality rate also increases [88, 89]. In a recent study, varying sizes of inhibition zones were noted during an examination of the effectiveness of Ag-NPs against different pathogenic bacteria. According to Al-Rajhi [90], the diameters recorded for *B. subtilis*, *S. aureus*, *E. coli*, and *P. aeruginosa* were 28.2 mm, 23.2 mm, 27.2 mm, and 28.4 mm, respectively. Our findings are consistent with previous research, as noted by Said et al. [91].

## Conclusion

This study demonstrates the successful green synthesis of silver nanoparticles (Ag-NPs) using *Withania coagulans* leaf extract, leveraging the plant’s phytochemicals as natural reducing and capping agents. The synthesized Ag-NPs were extensively characterized using UV–Vis, FT-IR, XRD, Raman, Zeta potential, DLS, SEM–EDS, and TGA–DSC, confirming their crystalline structure, stability, and an average particle size of approximately 37 nm. UV–Vis spectroscopy displayed a distinct surface plasmon resonance peak at 420 nm, indicative of Ag-NP formation. XRD analysis revealed high crystallinity with well-defined peaks corresponding to silver’s characteristic planes, while FT-IR spectra highlighted the functional groups responsible for the stabilization of Ag-NPs, including hydroxyl and carbonyl groups. SEM and HRTEM images confirmed irregular morphology with particle diameters averaging around 26.63 nm, while zeta potential analysis demonstrated moderate stability at − 21.4 mV. Biocompatibility assessments, including hemolysis and brine shrimp assays, indicated that the Ag-NPs exhibit moderate cytotoxicity within safe limits, suggesting potential for biomedical applications. Antimicrobial tests showed substantial inhibitory effects against *Klebsiella pneumoniae* and *Salmonella typhi*, with inhibition zones of 29 ± 0.01 mm and 28 ± 1.00 mm, respectively, demonstrating their efficacy against pathogenic microorganisms. This study pioneers the use of *Withania coagulans* leaf extract for the synthesis of Ag-NPs, presenting a comprehensive assessment of their structural, antioxidant, and antimicrobial properties, which could inform future applications in biomedical and agricultural domains. Overall, the findings underscore the versatility and potential of green-synthesized Ag-NPs in medicine, agriculture, and environmental applications. Future studies should further explore these nanoparticles’ specific applications and investigate the underlying mechanisms of their bioactivity in greater detail.

## Data Availability

The datasets generated during and/or analyzed during the current study are available from the corresponding author on reasonable request.
